# Editorial Comment on “Nomogram for Predicting the Survival Outcome of Cabazitaxel Treatment in Patients With Metastatic Castration‐Resistant Prostate Cancer: A Multi‐Institutional Analysis”

**DOI:** 10.1111/iju.70236

**Published:** 2025-09-17

**Authors:** Koji Hatano

**Affiliations:** ^1^ Department of Urology The University of Osaka Graduate School of Medicine Suita Japan

The treatment landscape for systemic therapy in metastatic castration‐resistant prostate cancer (mCRPC) has evolved rapidly with the emergence of novel therapies, expanding the range of treatment options available to clinicians [1]. The use of androgen receptor signaling inhibitors (ARSI) as first‐line therapy for metastatic prostate cancer is increasing; however, chemotherapy remains the mainstay of treatment for mCRPC. Since the TROPIC trial demonstrated that cabazitaxel (CBZ) improved survival in patients with mCRPC treated with docetaxel (DOC), sequential CBZ administration following DOC has become one of the preferred treatment options for mCRPC. Other treatment options for mCRPC after ARSI and DOC include 177Lu‐PSMA and Ra‐223 for symptomatic bone metastases, and poly (ADP‐ribose) polymerase inhibitors for patients with *BRCA* gene mutations. Compared with non‐chemotherapeutic agents, CBZ therapy frequently causes serious adverse events, such as severe neutropenia. Therefore, identifying patient subgroups most likely to benefit from CBZ remains an unmet clinical need.

Prognostic factors in mCRPC have been examined in several clinical studies, including patient factors such as performance status (PS); hematologic factors such as hemoglobin (Hb); tumor markers such as prostate‐specific antigen (PSA), lactate dehydrogenase (LDH), and alkaline phosphatase (ALP); and the extent of metastasis determined by imaging modalities. In 2003, prior to the DOC era, Halabi et al. developed a prognostic nomogram for mCRPC [2]. The prognostic factors included PS, Hb, PSA, LDH, ALP, Gleason score, and presence of visceral metastases. In 2014, Halabi et al. developed an updated prognostic nomogram for patients with mCRPC receiving DOC, incorporating PS, opioid analgesic use, albumin, Hb, PSA, LDH, ALP, and the site of disease [3]. Armstrong et al. developed a prognostic nomogram for patients with mCRPC receiving DOC, using the TAX327 trial cohort [4]. This model incorporated 10 prognostic factors: PS, pain, Hb, PSA, PSA doubling time, ALP, tumor grade, liver metastases, number of metastatic sites, and progression type.

Recently, Suzuki et al. developed a prognostic nomogram using a cohort of 345 patients with mCRPC who initiated CBZ therapy after DOC [5]. This nomogram predicts the one‐year survival probability using the following predictors: PS, Hb, PSA, PSA doubling time, LDH, presence of liver metastases, and radiographic progression during DOC. The C‐index for the Cox hazard model in internal and external validation was 0.72 and 0.67, respectively. In both cohorts, a significant difference in overall survival was observed between the risk groups defined by the total score calculated from the nomogram. This nomogram can predict survival outcomes after CBZ treatment in patients with mCRPC. The major limitation of this study was the heterogeneity of the mCRPC treatment sequence. Approximately 25% of patients did not receive ARSI treatment prior to CBZ. These patients may experience prolonged survival because of their response to ARSI after CBZ. Although further validation is required, this model has the potential to provide useful information for patients considering CBZ therapy.

## Author Contributions

**Koji Hatano:** writing – original draft and review.

## Conflicts of Interest

K.H. received honoraria for lecture fees from Astellas Pharma, AstraZeneca, Bayer Pharma, Janssen Pharma, Sanofi, and Takeda Pharmaceuticals. K.H. received research funding from Astellas Pharma.

## Linked Articles

Suzuki K, Hirata J, Ueki H, et al., “Nomogram for Predicting the Survival Outcome of Cabazitaxel Treatment in Patients With Metastatic Castration‐Resistant Prostate Cancer: A Multi‐Institutional Analysis” *International Journal of Urology* (2025), https://doi.org/10.1111/iju.70219.
